# Long-Term Results of Autologous Tooth Bone Grafting in Alveolar Cleft Reconstruction: A Retrospective Cohort Study

**DOI:** 10.3390/biomedicines13071735

**Published:** 2025-07-16

**Authors:** Tamás Würsching, Bence Mészáros, Eleonóra Sólyom, Bálint Molnár, Sándor Bogdán, Zsolt Németh, Krisztián Nagy

**Affiliations:** 1 Department of Oro-Maxillofacial Surgery and Stomatology, Semmelweis University, 1085 Budapest, Hungary; bogdan.sandor@semmelweis.hu (S.B.); nemeth.zsolt@semmelweis.hu (Z.N.); 2 Pediatric Center, Semmelweis University, 1083 Budapest, Hungary; meszaros.bence@semmelweis.hu (B.M.); nagy.krisztian@semmelweis.hu (K.N.); 3 Department Periodontology, Semmelweis University, 1088 Budapest, Hungary; solyom.eleonora@semmelweis.hu (E.S.); molnar.balint@semmelweis.hu (B.M.)

**Keywords:** alveolar bone grafting, autogenous tooth bone graft, 3D surgical planning, volumetric radiographic evaluation, unilateral cleft lip and palate

## Abstract

**Background/Objectives**: During alveolar cleft grafting, the use of autogenous cancellous bone harvested from the iliac crest is still considered the gold standard. Due to the risk of donor-site morbidity and excessive graft resorption, alternative grafting materials (e.g., intraoral bone, xenografts) are being tested. The aim of this study was to compare the efficacy of using an autologous tooth-derived graft material and iliac crest cancellous bone in the reconstruction of the alveolar cleft in patients with a unilateral cleft lip and palate. **Methods**: A total of 21 patients with a unilateral cleft lip and palate, who underwent alveolar bone grafting between 2020 and 2023 were included in the study. In 11 cases, the donor site was the iliac crest; in the rest of the cases, deciduous teeth were harvested, processed, and used as an autologous particulate graft material for alveolar reconstruction. The mean follow-up time was 30.0 months, CBCT scans were taken, and the results were compared based on the ranking system published by Stasiak et al. **Results**: The Wilcoxon signed-rank test showed that the amount of bone on the cleft side was significantly less than that on the contralateral non-cleft side (ATB: *p* = 0.002, iliac crest: *p* = 0.005). The Mann–Whitney U test showed that there were no significant differences in bone quantity on the cleft side between the two groups (U = 47.5, *p* = 0.617). **Conclusions**: The use of ATB might be a feasible alternative to autologous bone during alveolar cleft reconstruction. This type of graft shows long-term stability, which is comparable to the bone harvested from the iliac crest.

## 1. Introduction

Cleft lip and palate (CLP) are the most common congenital anomalies affecting the craniofacial region, characterized by the discontinuity of anatomical structures and varying degrees of tissue hypoplasia, affecting approximately 1 in every 700 children worldwide. The etiology is multifactorial, involving both genetic predispositions and environmental influences [1]. The comprehensive management of these patients presents significant clinical challenges and requires a coordinated, interdisciplinary approach, involving multiple medical and dental specialties. A critical component of this treatment protocol is alveolar bone grafting (ABG), which, usually combined with orthodontic treatment, plays a pivotal role in the anatomical and functional rehabilitation of the maxillary arch. The main objectives of ABG include reconstruction of the alveolar bony defect, restoration of arch continuity, support of the alar base, and closure of oronasal fistulae. The success of ABG is a crucial determinant in continuing orthodontic treatment and achieving favorable functional and aesthetic outcomes.

ABG is mostly performed during the mixed dentition phase, with the best outcomes observed when grafting is completed prior to the eruption of the cleft-side canine, usually between the ages of 9–12 years [2]. Early grafting allows the erupting tooth to stimulate and maintain the grafted bone, enhancing its integration and long-term stability. In cases where the lateral incisor is congenitally missing, an occurrence frequently seen in CLP patients, the success of the graft directly influences subsequent orthodontic decisions regarding space closure versus prosthetic replacement [3].

In terms of soft tissue management, the 4-flap technique described by Nordin and Abyholm is widely employed due to its effectiveness in achieving tension-free closure over the graft site. However, this method may not adequately address the limited width and thickness of keratinized gingiva adjacent to the cleft, potentially resulting in periodontal compromise and, in severe cases, eventual tooth loss. Moreover, if flap tension leads to wound dehiscence or suture failure, secondary healing and graft exposure can occur, increasing the risk of graft failure [4].

The preferred graft material for this procedure remains autologous bone, particularly from the iliac crest, as originally described by Boyne and Sands [5]. This donor site is favored because it provides ample cancellous bone and exhibits all three key osteogenic properties: osteoconduction, osteoinduction, and osteogenesis. Nevertheless, iliac crest harvesting is associated with notable donor-site morbidity, in addition to the risk of excessive graft resorption over time [6].

To address these limitations, recent research has focused on alternative grafting materials, such as allografts and synthetic substitutes, as well as biologically active adjuncts, including fibrin sealants, platelet-rich plasma (PRP), and leukocyte–platelet rich fibrin (L-PRF) [7,8,9,10]. These materials aim to reduce post-operative complications, enhance healing, and potentially improve the success rate of the grafting procedure [8,11]. One such material is auto-tooth bone, or ATB.

Auto-tooth bone is a bio-recycled material, originally developed in Korea, which is derived from extracted teeth and used as an autogenous bone graft after chemical and mechanical preparation. Unlike synthetic grafting materials, it possesses both osteoconductive and osteoinductive properties, promoting rapid new bone formation and remodeling. This graft material is composed of an incompletely demineralized dentin matrix, which contains type I collagen, similarly to alveolar bone, as well as bone morphogenetic proteins (BMPs) and calcium phosphate, all of which are key components of bone regeneration [12]. The mineral components of ATB graft materials consist of four stages (types) of calcium phosphate: hydroxyapatite, tricalcium phosphate, octacalcium phosphate, and amorphous calcium phosphate [13].When using ABG for guided bone regeneration, Kim et al. found that after 3 months, most ATB underwent resorption, and excellent bone healing and bone remodeling were observed, which occurred as a result of osteoinduction and osteoconduction. In regard to the histomorphometric analysis of the samples collected during the 3–6-month healing period, new bone formation was detected in 46–87% of the area of interest, and excellent bony remodeling was achieved [14]. The aim of the present study was to assess the long-term results of ABG using ATB derived from deciduous or supernumerary teeth. The hypothesis was that ATB might be a feasible alternative to autologous cancellous bone graft harvested from the iliac crest.

Although there is a general consensus regarding the necessity of the reconstruction of the alveolar cleft, the proper assessment of surgical outcomes remains challenging. A post-operative evaluation is crucial not only to measure the success of the procedure, but also to enable the early detection of graft resorption or displacement, which are among the most frequent complications of alveolar bone grafting.

The earliest assessment methods, such as the use of Bergland and Chelsea scales, relied on a two-dimensional approach, using intraoral X-rays [15]. Over time, with the emergence of computed tomography (CT) and especially cone-beam computed tomography (CBCT) technologies, several 3D evaluation methods have been developed to assess the outcomes of ABG. While 2D methods are limited by structural overlaps and cannot provide volumetric data, 3D techniques using CT overcome these limitations. Moreover, CBCT is often preferred over conventional CT for evaluating bone grafts due to its lower radiation exposure and its ability to accurately estimate the graft volume [16].

A preoperative CBCT scan also enables the 3D visualization of the alveolar bone defect morphology. The use of intraoral and extraoral scanners can also provide valuable information on the relevant soft tissues. Combining this data with specific software provides the opportunity for virtual surgical planning of both soft and hard tissue changes [17]. With the constant evolution and decreasing price of 3D printing, surgical templates and guides have become commonly used aids during maxillofacial surgery. Employing a 3D model for ABG to assess the complex anatomy of the defect can be beneficial during graft harvesting and can lead to a more accurate reconstruction. Auxiliary processes, such as rapid prototyping, augmented reality, and virtual reality, can also aid surgeons during the intervention and may reduce further complications.

## 2. Materials and Methods

### 2.1. Study Design and Patient Selection

Patients exhibiting a unilateral cleft lip and palate (UCLP), who had undergone alveolar bone grafting between 2020 and 2023, were recruited for this study. The enrolled patients were being treated at the Pediatric Center at Semmelweis University, Budapest. The study was approved by the university’s Regional and Institutional Committee of Science and Research Ethics (Approval Number: SE RKEB 251/2020). The study protocol was submitted and approved by the U.S. National Library of Medicine (www.clincaltrials.gov; trial registration number: NCT05971914). The research plan was compiled following the legislation in force and the Declaration of Helsinki from the World Medical Association (reference number: 23/2002. V.9.). Surgical interventions were undertaken with the understanding and written consent of each subject’s caregiver. The eligibility criteria were complete UCLP without other congenital deformities, ABG surgery using either a iliac crest cancellous bone graft or ATB from deciduous or supernumerary teeth, and CBCT imaging at least one year after grafting. The exclusion criteria were bilateral clefts, any previous attempt at bone grafting, the use of any other type of grafting material (e.g., xenografts, other donor site), and a lack of long-term follow-up CBCT. The STROBE checklist was used during the preparation of this manuscript.

### 2.2. Surgical Protocol

Every patient underwent the same preoperative preparation. In every case, the development of the cleft-side canines was considered during the planning of the surgery, as was the need for orthodontic treatment. All the patients included in the study required orthodontic maxillary expansion. The development of the canines was monitored using periapical X-rays. Only once the canine’s root development was between one-third and two-thirds of its expected final length was the decision made to proceed with surgery. At this point, CBCT scans were taken for surgical planning. All the enrolled patients were examined by two orthodontists to provide assurance that tooth removal was indicated, according to the patient’s age and orthodontic planning.

Based on the extent and morphology of the cleft, the desired shape of the nasoalveolar graft was designed using the freeware software 3D Slicer (www.slicer.org). Since every case was unilateral, the non-cleft side anatomy was used as a reference during graft planning. The upper border of the defect was taken as the lowest part of the piriform aperture on the non-affected side, while the lower border was taken as the cementoenamel junction of the neighboring teeth. The buccal and palatal borders were determined by a line connecting the most buccal and palatal points of the alveolar process, next to the defect [18]. This also provided the opportunity to determine the exact volume of the graft in cm^3^. This was a helpful addition in regard to planning the surgery because the maximum amount of material the ATB machine can produce is approximately 3 cm^3^, and the achievable volume of the ATB could be predicted based on the volume of the deciduous teeth.

**Figure 1:**
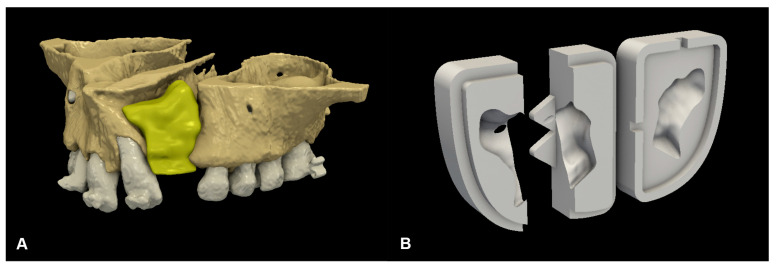
Preoperative virtual planning: (**A**) digital 3D plan of cleft reconstruction; and (**B**) CAD-modelled grafting template.

After planning, the model was exported as a standard tessellation language (STL) file. As the final step, a grafting template was constructed using an open-source computer-aided design (CAD) software, Blender (Blender Foundation, Amsterdam, The Netherlands), as described by Fabian et al. [19]. The designed parts were manufactured with a stereolithography (SLA) 3D printer (Phrozen Shuffle XL; Phrozen, Hsinchu City, Taiwan), using class I biocompatible, surgical-grade resin (Dental SG; NextDent BV, Soesterberg, The Netherlands) (Figure 1).

**Figure 2:**
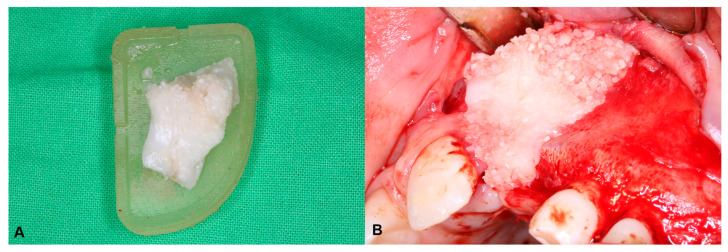
Hard-tissue reconstruction of the alveolar cleft: (**A**) the mixture of fibrin sealant and ATB particles was shaped by the 3D-printed grafting template; and (**B**) reconstruction of the alveolar defect using the ATB graft.

In cases where the teeth were used for ATB, they were extracted under general anesthesia during the same surgery as when the ABG was performed. The extracted teeth were prepared immediately after removal, according to the Bonmaker^®^ protocol (Korea Dental Solution Co., Ltd., Busan, Republic of Korea). Ready-to-use ATB was mixed with a fibrin sealant (Tisseel^®^; Baxter, Glenview, IL, USA) in the digitally planned and 3D-printed grafting templates to obtain a sticky ATB graft that matched the shape and extent of the bony defect. The pre-shaped ATB graft was then inserted into the cleft defect. The detailed surgical protocol is described in the authors’ previous article [20] (Figure 2). In cases where the number of extractable teeth was not enough for the preparation of a sufficient amount of ATB, cancellous bone from the iliac crest was used instead.

In these cases, a two-team approach was chosen to reduce the operation time. The skin was draped and prepared, leaving the anterior superior iliac spine (ASIS) and the anterior iliac crest exposed. Before incision, local anesthetic was administered (Lidocaine with 0.2% Adrenaline). The skin incision was usually about 2 cm long and respected the patient’s relaxed skin tension lines. During the early adolescent age, when alveolar bone grafting is performed, the iliac crest is usually still covered by a layer of cartilage, which is incised in a rectangular shape. This piece was elevated medially, hinged on the inner edge of the iliac crest. Curettes were used to harvest cancellous bone chips. These were inserted into the 3D-printed mold and were mixed with Tisseel fibrin glue, as described above, to give the graft better formal stability. After the setting of the fibrin glue, the graft was removed from the mold, preserving the shape designed preoperatively, and was ready to be transferred to the cleft site. A collagen sponge was inserted into the donor area to promote hemostasis. The retracted cartilage was repositioned, and the wound was closed in layers. No drains were used [21].

### 2.3. Radiographic Image Acquisition and Assessment of Surgical Outcomes

CBCT scans were acquired, with a 10 × 8 cm field of view and a 200 µm voxel size, 110 kV, 5 mAs, using a Newtom VGI EVO^®^ device (Cefla S.C., Imola, Italy). The Digital Imaging and Communications in Medicine (DICOM) datasets were imported into the image processing software, Radiant (Medixant, Poznan, Poland, https://www.radiantviewer.com).

Assessment of the surgical outcomes was performed following the protocol described by Stasiak et al. [22], namely using a CBCT-based qualitative scoring system that is based on the presence and the relative dimensions of the bony bridge between the medial and lateral side of the alveolar cleft in four different vertical positions along the central incisor.

The evaluation began by reorienting the images along the long axes of the central incisors on each respective side. The cementoenamel junction (CEJ) served as the reference point for determining four measurement levels: 3 mm, 5 mm, 7 mm, and 9 mm. The CEJ was defined as the most apical point of the enamel in the midsagittal section of the incisor.

**Figure 3:**
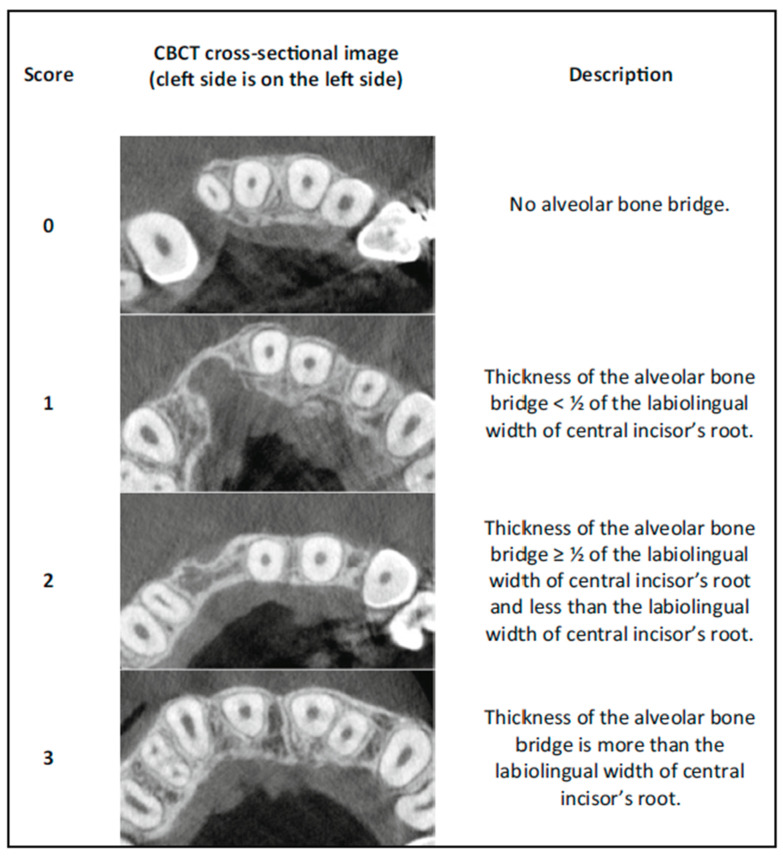
Horizontal scale for bone fill assessment, as described by Stasiak et al. [22].

The next step involved examining the presence or absence of a bone bridge through the continuous analysis of the region. Next, the alveolar bone was ranked at the designated levels by identifying the narrowest points between the central incisors and canines, using a horizontal scale, as follows:

- 0 = No alveolar bone bridge;
- 1 = The thickness of the alveolar bone bridge < ½ of the labiolingual width of the central incisor’s root;
- 2 = The thickness of the alveolar bone bridge ≥ ½ of the labiolingual width of the central incisor’s root and less than the labiolingual width of the central incisor’s root;
- 3 = The thickness of the alveolar bone bridge is at least the labiolingual width of the central incisor’s root (Figure 3).

To assess the overall bone quality, the rank scores for each level were summed up for each side. The total score was interpreted using an interval scale, as follows:

- 0 = Failure;
- 1–4 = Poor outcome;
- 5–8 = Moderate outcome;
- 9–12 = Good outcome.

A score of 0 was assigned when no bone bridge was present. In cases where a narrow bone bridge existed but fell between or above the designated measurement levels (3, 5, 7, or 9 mm), a modified evaluation was applied. This involved conducting scoring at the actual bone bridge level and attributing the score to the nearest standard level.

Additionally, for cases with severe root resorption of the central incisors, classification using the horizontal scale was still performed at the 9 mm level. However, the assessment was adjusted by comparing the root width, measured 0.5 mm below the apex, to provide a more accurate evaluation.

### 2.4. Statistical Analysis

The data was collected in Microsoft Excel (Microsoft, Washington, CA, USA). Statistical analyses were performed using SPSS version 30.0. (IBM Corporation, Redmond, WA, USA). The Kappa correlation coefficient with linear weights was used for the intra-rater and inter-rater reproducibility measurements. To test the normality of the acquired data, the Shapiro–Wilk test was used. The non-parametric Wilcoxon signed-rank test was used for a comparison of the cleft-side and non-cleft-side bone volume. The non-parametric Mann–Whitney U test was used to compare the Stasiak score and the patient’s age at the time of surgery of the iliac crest group and the ATB group. Student’s t-test was used to compare the initial defect volume of the two groups. The correlation between the bone defect volume and the outcome score was analyzed separately for the iliac crest and ATB groups, using Spearman’s rank correlation.

## 3. Results

### 3.1. Patient Demographics and Descriptive Statistics

**Figure 4:**
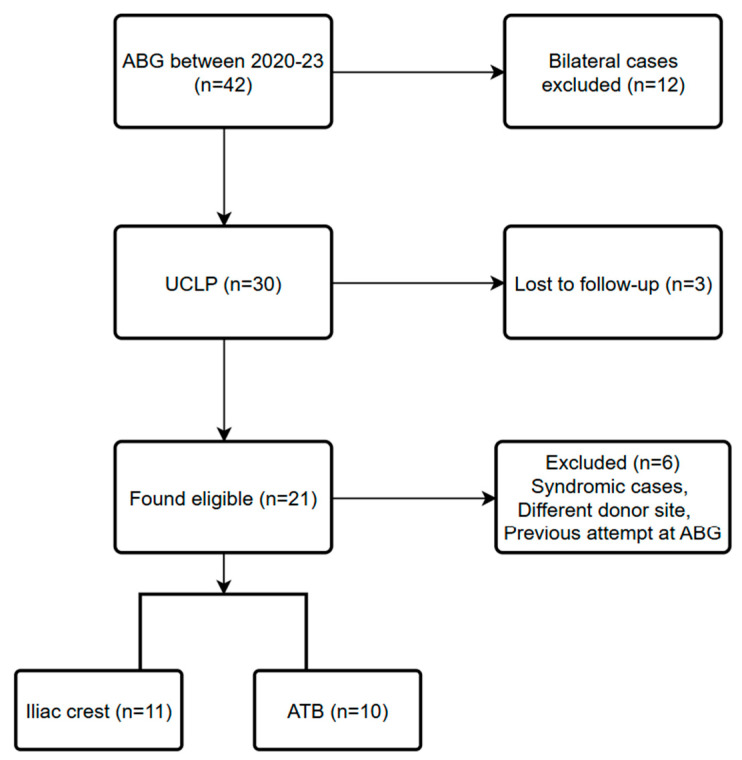
STROBE flowchart.

During the period examined, a total of 42 ABG surgeries were performed in our department. In 12 cases, the defect was bilateral; in 30 cases, the defect was unilateral. In 14 cases, an ATB graft was used; in 25 cases, the donor site was the iliac crest; in three cases, the donor site was the chin. Long-term CBCT data was available for 39 patients; three patients were lost to follow-up. Twenty-one participants exhibiting UCLP were found to be eligible and were enrolled in the current study (Figure 4.). Thirteen patients were male, eight were female. Their age at the time of surgery was between 8 and 14 years; the mean age was 10.4 ± 1.7 years. Sixteen defects were located on the left side and five on the right side. In eleven cases, the donor site for the graft was the iliac crest. In the other 10 cases, a total of 53 deciduous teeth and 4 supernumerary teeth were extracted and utilized for the preparation of the ATB graft. In these cases, the patient’s own teeth provided enough material for the proper reconstruction of the alveolar defect, using an average of 5.3 ± 2.26 teeth per patient. The mean follow-up time was 30 ± 13.1 months.

The mean volume of the initial defect in the iliac crest group was 0.927 cm^3^ (SD = 0.316 cm^3^) and, in the ATB group, it was 1.176 cm^3^ (SD = 0.449 cm^3^). The mean age during the time of surgery in the iliac crest group was 10.9 years (SD = 2.1 years) and, in the ATB group, it was 9.9 years (SD = 1.2 years). There was no obvious difference between the occurrence of early (dehiscence) or late (fistula) complications. In one patient, a severe periodontal defect also developed on the distal surface of tooth 21, which led to extraction. In the ATB group, canine eruption did not happen in two cases during the observed period, whereas no such problems were observed in the other group (Table 1 and Table 2).

### 3.2. Radiographical Assessment

The measurements on both the cleft and non-cleft sides were performed three times in all the patients and, during each instance, all the 168 sites were measured. The first author performed the measurements twice during a 4-week period, and the second author performed the measurements only once. Finally, a consensus reading was performed by both authors.

On the cleft side of the iliac crest group, fifteen sites were classified as 0, four sites as 1, ten sites as 2, and thirteen sites as 3. On the cleft side of the ATB group, twelve sites were classified as 0, five sites as 1, ten sites as 2, and thirteen sites as 3.

On the control side, no 0 or 1 scores were obtained. There were six sites classified as 2 and seventy-eight sites as 3.

**Figure 5:**
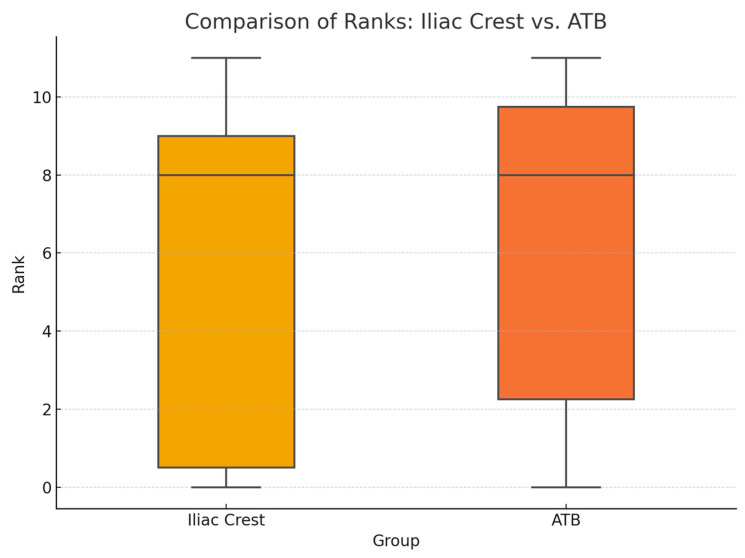
Comparison of the mean of the rank scores on the cleft side.

**Figure 6:**
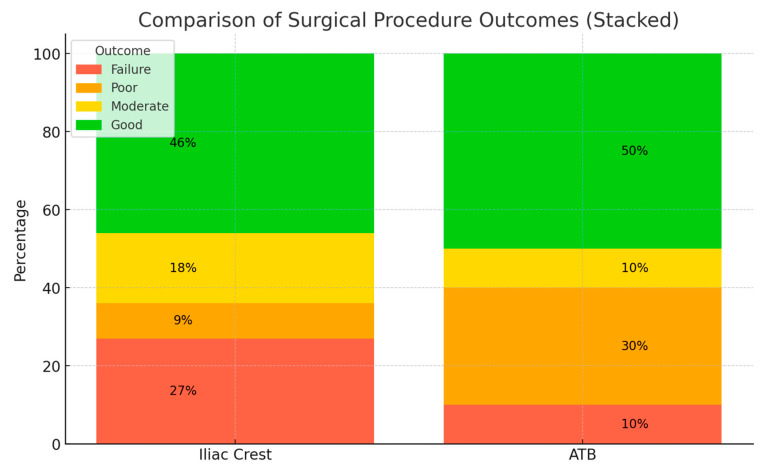
Comparison of surgical procedure outcomes.

The surgical outcome showed a high level of variability. The mean total score on the cleft side in the iliac crest group was 5.8 (SD: ±4.6); in the ATB group, the mean total score on the cleft side was 6.4 (SD: ±4.2). (Figure 5). On the non-cleft side, the mean total score was 11.7 (SD: ±0.5). In the iliac crest group, the results showed 27% failure, 9% poor, 18% moderate, and 46% good results of the surgical procedure. In the ATB group, the results were 10% failure, 30% poor, 10% moderate, and 50% good. The alveolar bone was classified as good in all the patients on the non-cleft side (Figure 6).

### 3.3. Statistical Analysis

In regard to all the statistical measurements, a 95% confidence interval was adopted. The Wilcoxon signed-rank test showed statistically significant (ATB: *p* = 0.002, iliac crest: *p* = 0.005) differences between the cleft- and non-cleft-side measurements, which did not depend on the type of graft being used. The linearly weighted Kappa coefficient result was 0.85 for intra-rater and 0.82 for inter-rater reproducibility. These results showed the excellent reproducibility of the method presented. The Mann–Whitney U test showed no significant differences between either the Stasiak scores (U = 47.5, *p* = 0.617) or the age at the time of surgery (U = 67.5, *p* = 0.388) of the two groups.

A comparison of the defect volumes between the two groups was performed using an independent sample Student’s t-test. Prior to the analysis, the assumptions for parametric testing were evaluated: Shapiro–Wilk tests confirmed that the volume data were normally distributed in both the iliac crest (*p* = 0.61) and ATB (*p* = 0.86) groups, and Levene’s test indicated the homogeneity of the variances (*p* = 0.36). The t-test revealed no statistically significant difference in the mean defect volume between the iliac crest group and the ATB group (t(df) = −1.48, *p* = 0.155).

**Figure 7:**
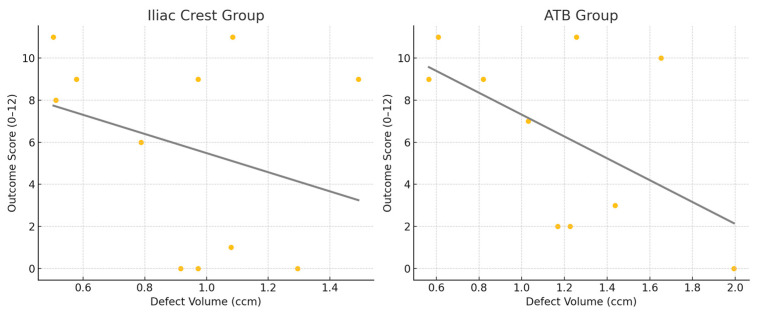
Correlation between initial defect size and outcome score.

The correlation between the bone defect volume and the outcome score was analyzed separately for the iliac crest and ATB groups, using Spearman’s rank correlation. In the iliac crest group, a weak negative correlation was observed (ρ = −0.19, *p* = 0.58), while the ATB group showed a moderate negative correlation (ρ = −0.36, *p* = 0.31). However, neither correlation reached statistical significance (*p* > 0.05). Scatter plots with trendlines are used to illustrate the inverse relationship between the defect volume and outcome score in both groups (Figure 7).

## 4. Discussion

In the present study, the long-term outcomes of using ATB for alveolar bone grafting in patients with a cleft lip and palate were compared to the results achieved by using the iliac crest as a donor site.

Auto-tooth bone grafts can effectively restore damaged alveolar bone using natural bone tissue and offer solutions to several challenges associated with other grafting materials, such as insufficient osteoinduction, a lack of osteoconduction, sterilization concerns, and the risk of disease transmission. This type of graft is commonly used in preprosthetic and periodontal regenerative surgery [23,24].

Although there are articles describing the use of ATB in cleft alveolar bone grafting that have been published previously, they are singular case reports [25,26,27]. The authors have previously published the short-term results of a case series involving ATB, which showed that although after three months graft resorption could be observed, the amount that was lost was on a par with what could be expected from the use of bone from the iliac crest, according to the literature [20]. Long-term results on the use of ATB in alveolar bone grafting have not yet been published.

Nowadays, there are several similar devices available that are based on the same principle, namely preparing an autologous graft from the patient’s tooth. In the present study, the Bonmaker^®^ device (Korea Dental Solution Co., Ltd., Busan, Korea) was used, in the cases where the number of deciduous teeth was adequate. For the rest of the patients, the donor site was the iliac crest, in which a two-team approach was adopted to optimize the surgical time. To achieve the proper amount of graft material according to the preoperative planning, an average of 5.3 ± 2.26 teeth per patient were used. As the early loss of deciduous teeth can lead to complications during eruption of the patient’s permanent teeth, our aim was to avoid unnecessary extractions. This means, however, that the roots of these teeth were usually partially or completely resorbed, meaning less graft volume per tooth was extracted. This also means that the amount of graft that can be prepared through the use of this method is usually only sufficient for unilateral clefts, so no bilateral cases were treated this way, and they were excluded from this study.

Apart from the lack of an extraoral donor site, the ATB protocol offers two other notable advantages: (i) Approximately half of the process, around 20 min, is fully automated and is carried out by the Bonmaker device without requiring any user input. This automation allows medical staff to focus on other tasks during surgery. (ii) The preparation of the ATB graft is simple and does not require a highly specialized physician. Nurses are fully capable of managing the graft preparation process independently.

The estimated costs associated with the use of ATB are approximately EUR 35–40, depending on the type of device and differences in local prices, and the price of the device itself is also a one-time additional cost. While this contributes to the overall surgical expenses, the costs are not considered extremely high. Moreover, the use of this approach eliminates the need for an extraoral donor site, leading to shorter hospital stays.

After using the iliac crest as a donor site, Brudnicki et al. [28] reported an average hospital stay of 2.9 days, ranging from 1 to 8 days, with most patients being discharged on the second or third day after surgery. In higher-income countries, where hospital stays are more costly, the reduced length of hospitalization associated with the ATB protocol could potentially lower the overall treatment costs. However, a detailed cost-effectiveness analysis was beyond the scope of this study.

Alveolar bone grafting is considered successful when it achieves sufficient bone filling in the alveolar cleft. The eruption of adjacent teeth, particularly the canine, through the grafted bone is also a critical indicator of treatment success. Additionally, the lack of oronasal fistulae, healthy periodontal tissues around teeth adjacent to the graft site, and the maintenance of alveolar bone stability and continuity over time are also essential criteria [29]. A recent meta-analysis by Jahanbin et al. showed that the total percentage of bone filling after 1 year, according to CBCT, was about 63.38% [30].

Due to the limited sample size in this study, it is not possible to conclude whether there is a significant difference between the rate of early or late complications between the two groups. However, the fact that canine eruption in the iliac crest group happened in every case and, in the ATB group, there were two cases out of ten where it did not, needs to be addressed. This must be followed on a longer-term basis and in regard to a larger sample size, as the failure of the canines to spontaneously erupt leads to further surgical interventions, which is a factor that must be considered when choosing ATB as a potential graft material.

CBCT is increasingly used to evaluate the results of ABG, due to its ability to provide detailed 3D imaging with lower radiation than conventional CT, replacing conventional methods based on 2D X-rays. While several CBCT-based methods exist, none have achieved universal recognition [31,32]. Volumetric assessments quantify bone fill (BF), but lack spatial specificity, limiting their value in regard to clinical decision making. Grading scales using 2D measurements at vertical levels, such as those by Soumalainen and Liu [33], offer partial localization, but are affected by the dental eruption status and have limited generalizability.

Assessment approaches are generally classified as continuous (e.g., bone volume or resorption rates) or categorical (e.g., bone height/width). Continuous methods, while quantitative, lack standardized clinical thresholds and fail to identify critical bone deficits impacting orthodontic outcomes.

Stasiak et al. introduced a more advanced, clinically applicable method that divides the cleft into defined zones, enabling the precise localization of bone presence and resorption. It uniquely considers the impact of bone loss on adjacent teeth and also considers root resorption. This spatially detailed approach supports more informed decisions about regrafting and orthodontic planning, representing a major improvement to CBCT-based ABG evaluations. This method was implemented in the current study, and proved to be reliable and reproducible, with excellent rates of intra- and inter-rater reliability.

The results show that there are no significant differences between the outcomes of the two groups based on the Stasiak scale and, in both groups, at least 60% of the cases fell into the moderate or good category, meaning no regrafting was deemed necessary. In terms of the other cases, approximately 40% of the cases where the outcome fell into the poor or the failure category, regrafting or prosthetic rehabilitation needed to be considered on an individual basis.

## 5. Conclusions

Alveolar bone grafting in patients with a cleft lip and palate remains a challenging task, with no currently known method or type of graft material providing a substantially higher rate of success than the others. The results of this study indicate that an ATB graft is a safe and predictable graft material with long-term volumetric stability, which may be utilized in alveolar cleft reconstruction. The main limitation of this study is the relatively small sample size, which causes us to be cautious about generalizing the results in regard to cleft surgery. The use of ATB, however, is well-documented, and is already deemed a reliable material in preprosthetic and periodontal surgery. Another limiting factor is that the long-term results were not compared to the initial defect size in a volume-based manner, but instead the categorical scaling system developed by Stasiak et al. was used. This system, however, is straightforward, reliable, and aids the user in regard to clinical decision making.

Although the sample size was small, no significant differences were observed compared to the current gold standard of grafts, the iliac crest; however, the bone volume at the reconstructed site was still significantly less than on the healthy contralateral side, regardless of the type of graft used. The application of an ATB graft requires additional training and equipment, but the good clinical results and the lack of an extraoral donor site may compensate for this and lead to this method being recommended for the alveolar reconstruction of patients with a cleft lip and palate. However, due to the limited amount of graft that can be prepared from deciduous or supernumerary teeth in this patient population, the method presented is recommended by the authors for use in small-to-medium size unilateral alveolar defects. Further studies with larger sample sizes are of course necessary to support the findings in the present study.

## Figures and Tables

**Table 1:** Descriptive statistics of the iliac crest group.

Iliac Crest	Vdefect (cm^3^)	Dehiscence	Fistula	Canine	Stasiak Score	Age at Surgery (years)	Follow-Up (months)
Patient 1	1.085	no	no	erupted	11	9	58
Patient 2	1.492	yes	fistula	erupted	9	14	24
Patient 3	0.973	no	no	erupted	0	14	41
Patient 4	0.578	no	no	erupted	9	12	36
Patient 5	0.504	yes	no	erupted	11	13	14
Patient 6	0.916	no	no	erupted	0	10	49
Patient 7	0.973	no	no	erupted	9	8	18
Patient 8	0.788	no	fistula	erupted	6	10	24
Patient 9	0.512	no	no	erupted	8	9	52
Patient 10	1.295	no	no	erupted	0	10	12
Patient 11	1.079	yes	no	erupted	1	11	56

**Table 2:** Descriptive statistics of the ATB group.

ATB	Vdefect (cm^3^)	Dehiscence	Fistula	Canine	Stasiak Score	Age at Surgery (years)	Follow-Up (months)
Patient 12	1.994	no	no	not erupted	0	10	28
Patient 13	0.819	no	no	erupted	9	9	25
Patient 14	1.653	yes	no	erupted	10	8	20
Patient 15	1.03	no	no	erupted	7	11	20
Patient 16	1.226	yes	21 perio	erupted	2	11	26
Patient 17	0.609	no	no	erupted	11	11	28
Patient 18	1.438	no	no	not erupted	3	8	29
Patient 19	1.256	no	no	erupted	11	10	25
Patient 20	1.169	no	fistula	erupted	2	11	25
Patient 21	0.565	no	no	erupted	9	10	23

## Data Availability

The data presented in this study is available on request from the corresponding author. Measurements based on CBCT can be presented after patient anonymization, the raw CBCT data is not available due to patient privacy.

## Abbreviations

The following abbreviations are used in this manuscript:

CLP	Cleft lip and palate
ABG	Alveolar bone grafting
ATB	Auto-tooth bone
PRP	Platelet-rich plasma
L-PRF	Leukocyte–platelet rich fibrin
BMP	Bone morphogenic protein
HA	Hydroxyapatite
CT	Computed tomography
CBCT	Cone-beam computed tomography
UCLP	Unilateral cleft lip and palate
STL	Standard tessellation language
CAD	Computer-aided design
SLA	Stereolithography
CEJ	Cementoenamel junction

## References

[1] Acs L., Nemes B., Nagy K., Acs M., Banhidy F., Rozsa N. (2024). Maternal factors in the origin of cleft lip/cleft palate: A population-based case-control study. Orthod. Craniofacial Res..

[2] Vandersluis Y.R., Fisher D.M., Stevens K., Tompson B.D., Lou W., Suri S. (2020). Comparison of dental outcomes in patients with nonsyndromic complete unilateral cleft lip and palate who receive secondary alveolar bone grafting before or after emergence of the permanent maxillary canine. Am. J. Orthod. Dentofac. Orthop..

[3] Fahradyan A., Tsuha M., Wolfswinkel E.M., Mitchell K.S., Hammoudeh J.A., Magee W. (2019). Optimal Timing of Secondary Alveolar Bone Grafting: A Literature Review. J. Oral. Maxillofac. Surg..

[4] Åbyholm F.E., Olav B., Semb G. (1981). Secondary Bone Grafting of Alveolar Clefts: A Surgical/Orthodontic Treatment Enabling a Non-prosthodontic Rehabilitation in Cleft Lip and Palate Patients. Scand. J. Plast. Reconstr. Surg..

[5] Boyne P.J., Sands N.R. (1972). Secondary bone grafting of residual alveolar and palatal clefts. J. Oral Surg..

[6] Dimitriou R., Mataliotakis G.I., Angoules A.G., Kanakaris N.K., Giannoudis P.V. (2011). Complications following autologous bone graft harvesting from the iliac crest and using the RIA: A systematic review. Injury.

[7] Brezulier D., Chaigneau L., Jeanne S., Lebullenger R. (2021). The Challenge of 3D Bioprinting of Composite Natural Polymers PLA/Bioglass: Trends and Benefits in Cleft Palate Surgery. Biomedicines.

[8] Ellapakurthi P., Reddy G.S.P. (2021). The effectiveness of mineralized plasmatic matrix in the closure of alveolar clefts with volumetric assessment. Regen. Med. Res..

[9] Janssen N.G., Weijs W.L., Koole R., Rosenberg A.J., Meijer G.J. (2014). Tissue engineering strategies for alveolar cleft reconstruction: A systematic review of the literature. Clin. Oral. Investig..

[10] Wu C., Pan W., Feng C., Su Z., Duan Z., Zheng Q., Hua C., Li C. (2018). Grafting materials for alveolar cleft reconstruction: A systematic review and best-evidence synthesis. Int. J. Oral. Maxillofac. Surg..

[11] Leal C.R., de Carvalho R.M., Ozawa T.O., de Almeida A.M., da Silva Dalben G., da Cunha Bastos J.C., Garib D.G. (2019). Outcomes of Alveolar Graft With Rhbmp-2 in CLP: Influence of Cleft Type and Width, Canine Eruption, and Surgeon. Cleft Palate Craniofacial J..

[12] Hussain A.A., Al-Quisi A.F., Abdulkareem A.A. (2023). Efficacy of Autogenous Dentin Biomaterial on Alveolar Ridge Preservation: A Randomized Controlled Clinical Trial. Biomed Res. Int..

[13] Kim Y.K., Kim S.G., Yun P.Y., Yeo I.S., Jin S.C., Oh J.S., Kim H.J., Yu S.K., Lee S.Y., Kim J.S. (2014). Autogenous teeth used for bone grafting: A comparison with traditional grafting materials. Oral Surg. Oral Med. Oral Pathol. Oral Radiol..

[14] Kim Y.K., Kim S.G., Byeon J.H., Lee H.J., Um I.U., Lim S.C., Kim S.Y. (2010). Development of a novel bone grafting material using autogenous teeth. Oral Surg. Oral Med. Oral Pathol. Oral Radiol. Endodontology.

[15] Lorenzoni D.C., Janson G., Bastos J.C., Carvalho R.M., Bastos J.C., de Cassia Moura Carvalho Lauris R., Henriques J.F., Ozawa T.O. (2017). Evaluation of secondary alveolar bone grafting outcomes performed after canine eruption in complete unilateral cleft lip and palate. Clin. Oral Investig..

[16] Lemberger M., Benchimol D., Pegelow M., Jacobs R., Karsten A. (2024). Validation and comparison of 2D grading scales and 3D volumetric measurements for outcome assessment of bone-grafted alveolar clefts in children. Eur. J. Orthod..

[17] Major M., Meszaros B., Wursching T., Polyak M., Kammerhofer G., Nemeth Z., Szabo G., Nagy K. (2024). Evaluation of a Structured Light Scanner for 3D Facial Imaging: A Comparative Study with Direct Anthropometry. Sensors.

[18] Wursching T., Kesztyus A., Pottel L., Swennen G., Nagy K. (2025). Comparison of two methods for segmentation of the nasoalveolar defect and design of a three-dimensional surgical template in patients with cleft lip and palate: A retrospective study. Int. J. Oral Maxillofac. Surg..

[19] Fabian Z., Kadar K., Patonay L., Nagy K. (2019). Application of 3D Printed Biocompatible Plastic Surgical Template for the Reconstruction of a Nasoalveolar Cleft with Preoperative Volume Analysis. Mater. Plast..

[20] Molnar B., Wursching T., Solyom E., Palvolgyi L., Radoczy-Drajko Z., Palkovics D., Nagy K. (2024). Alveolar cleft reconstruction utilizing a particulate autogenous tooth graft and a novel split-thickness papilla curtain flap—A retrospective study. J. Cranio-Maxillofac. Surg..

[21] Kesztyus A., Wursching T., Nemes B., Palvolgyi L., Nagy K. (2022). Evaluation of 3D visualization, planning and printing techniques in alveolar cleft repair, and their effect on patients’ burden. J. Stomatol. Oral Maxillofac. Surg..

[22] Stasiak M., Wojtaszek-Slominska A., Racka-Pilszak B. (2021). A novel method for alveolar bone grafting assessment in cleft lip and palate patients: Cone-beam computed tomography evaluation. Clin. Oral Investig..

[23] Radoczy-Drajko Z., Windisch P., Svidro E., Tajti P., Molnar B., Gerber G. (2021). Clinical, radiographical and histological evaluation of alveolar ridge preservation with an autogenous tooth derived particulate graft in EDS class 3–4 defects. BMC Oral Health.

[24] Solyom E., Szalai E., Czumbel M.L., Szabo B., Vancsa S., Mikulas K., Radoczy-Drajko Z., Varga G., Hegyi P., Molnar B. (2023). The use of autogenous tooth bone graft is an efficient method of alveolar ridge preservation—Meta-analysis and systematic review. BMC Oral Health.

[25] Jeong K.-I., Lee J., Kim K.-W., Um I.-W., Hara S., Mitsugi M., Kim Y.-K. (2013). Alveolar Cleft Reconstruction Using Chin Bone and Autogenous Tooth Bone Graft Material: Reports of 5 Cases. J. Korean Dent. Sci..

[26] Datarkar A., Bhawalkar A. (2020). Utility of tooth as an autogenous graft material in the defects of alveolar cleft—A novel case report. J. Oral Biol. Craniofacial Res..

[27] Hara S., Mitsugi M., Kanno T., Tatemoto Y. (2013). Bone transport and bone graft using auto-tooth bone for alveolar cleft repair. J. Craniofacial Surg..

[28] Brudnicki A., Regulski P.A., Sawicka E., Fudalej P.S. (2021). Alveolar Volume Following Different Timings of Secondary Bone Grafting in Patients with Unilateral Cleft Lip and Palate. A Pilot Study. J. Clin. Med..

[29] Yu X., Huang Y., Li W. (2022). Correlation between alveolar cleft morphology and the outcome of secondary alveolar bone grafting for unilateral cleft lip and palate. BMC Oral Health.

[30] Jahanbin A., Kamyabnezhad E., Raisolsadat M.A., Farzanegan F., Bardideh E. (2022). Long-Term Stability of Alveolar Bone Graft in Cleft Lip and Palate Patients: Systematic Review and Meta-Analysis. J. Craniofacial Surg..

[31] Kumar A., Batra P., Sharma K., Raghavan S., Talwar A., Srivastava A., Sood S.C. (2022). A Three-Dimensional Scale for the Qualitative and Quantitative Assessments of Secondary Alveolar Bone Grafting (SABG) in Unilateral Cleft Lip and Palate Patients Using Cone-Beam Computed Tomography (CBCT). Indian. J. Plast. Surg..

[32] Shaheen E., Danneels M., Doucet K., Dormaar T., Verdonck A., Cadenas de Llano-Perula M., Willems G., Politis C., Jacobs R. (2022). Validation of a 3D methodology for the evaluation and follow-up of secondary alveolar bone grafting in unilateral cleft lip and palate patients. Orthod. Craniofacial Res..

[33] Suomalainen A., Aberg T., Rautio J., Hurmerinta K. (2014). Cone beam computed tomography in the assessment of alveolar bone grafting in children with unilateral cleft lip and palate. Eur. J. Orthod..

